# Fabrication and Partial Characterization of Silver Nanoparticles From Mangrove (Avicennia marina) Leaves and Their Antibacterial Efficacy Against Oral Bacteria

**DOI:** 10.7759/cureus.52131

**Published:** 2024-01-11

**Authors:** Muskan Soni, Sivaperumal Pitchiah, Vasugi Suresh, Pasiyappazham Ramasamy

**Affiliations:** 1 Department of Physiology, Saveetha Dental College & Hospitals, Basic Medical Sciences, Saveetha Institute of Medical and Technical Sciences (SIMATS) Saveetha University, Chennai, IND; 2 Department of Prosthodontics, Saveetha Dental College & Hospitals, Saveetha Institute of Medical and Technical Sciences (SIMATS) Saveetha University, Chennai, IND; 3 Polymer Research Laboratory (PR Lab), Centre for Marine Research and Conservation, Saveetha Dental College & Hospitals, Saveetha Institute of Medical and Technical Sciences (SIMATS) Saveetha University, Chennai, IND

**Keywords:** good health and well-being, avicennia marina, anti-bacterial, silver nanoparticles, innovative, biosynthesis

## Abstract

Background: Scientists are currently investigating ecologically sound and enduring techniques for nanoparticle production. Utilizing natural sources such as plant extracts provides an environmentally friendly and economically efficient method. *Avicennia marina*, also referred to as the gray mangrove, is predominantly located in coastal regions. The leaves of this plant may contain bioactive metabolites that can be used to synthesize nanoparticles.

Objectives: This study aimed to synthesize silver nanoparticles (AgNPs) using *A. marina* leaf extract and subsequently assess their antibacterial properties against oral pathogens.

Materials and methods: The present research involved the successful synthesis of AgNPs using an environmentally sustainable method employing the leaf extract of *A. marina.* The reduction of Ag ions to AgNPs was confirmed using UV-visible spectroscopy. This analytical technique revealed the presence of a distinct surface plasmon resonance peak at approximately 420 nm, which is indicative of the formation of AgNPs. Fourier transform infrared spectroscopy (FTIR) operating within the frequency range of 500-3500 cm^−1^ and scanning electron microscopy (SEM) morphology of the image indicated agglomeration of the nanoparticles, with distinct particles ranging from 10 to 20 nm and dense rod-shape, which was carried out from Saveetha Dental College and Hospitals, Saveetha Institute of Medical and Technical Sciences (SIMATS), Chennai, Tamil Nadu, India. In energy-dispersive spectroscopy (EDS), a strong signal and maximum formation percentage were received at 42.7%, assigned to the element silver.

Results: AgNPs showed significant antibacterial efficacy against both gram-positive bacteria, including *Staphylococcus aureus* and *Streptococcus mutans*, and gram-negative bacteria, such as *Klebsiella *sp. In general, the use of *A. marina* leaf extract for the green synthesis of AgNPs is a viable and environmentally friendly approach for producing nanoparticles that exhibit favorable biological properties. Consequently, these nanoparticles hold considerable appeal as potential candidates for a range of biomedical applications, particularly as antibacterial agents.

Conclusion: The synthesis of AgNPs using *A. marina* leaf extract shows great potential in the field of creating nanomaterials that are compatible with biological systems and is promising for a wide range of clinical applications. Nevertheless, it is imperative to conduct comprehensive scientific research and rigorous clinical trials to effectively apply these discoveries to real-world medical interventions, while prioritizing patient safety and therapeutic effectiveness.

## Introduction

Nanoscience is a highly influential and inspirational scientific field that has yielded a wide range of unique cost-effective applications. Nanotechnology research plays a significant role in the advancement of multiple sectors of the medical industry. Nanoparticles (NPs) possess distinct characteristics attributed to their diminutive dimensions, which typically range from 1 to 100 nm, expansive surface areas, and surface properties [1]. Silver is widely recognized as the most economically advantageous precious metal for the synthesis and fabrication of NPs and nanomaterials. These nanoparticles are widely recognized for their antibacterial, antiviral, antifungal, and antioxidant properties and remarkably improved physicochemical characteristics compared to those of larger-scale materials. These enhanced features include the optical, thermal, electrical, and catalytic properties. Prior to the discovery that microorganisms are responsible for causing infections, silver (Ag) was extensively recognized for its medicinal and therapeutic properties. Previous studies have demonstrated the significant inhibitory and bactericidal effects of silver products [2,3]. The increasing need for AgNPs necessitates the exploration of ecologically sustainable synthetic methods. AgNPs can be synthesized using chemical, physical, or biological methods. Chemical treatments predominantly rely on chemical reduction processes involving interactions between Ag+ ions and both organic and inorganic substances. Physical approaches rely on the utilization of the evaporation-condensation technique as well as laser ablation of silver bulk material in a solution [4,5].

The use of plants for the synthesis of AgNPs is becoming increasingly popular owing to their environment-friendly characteristics, ease of access, cost-effectiveness, simplicity of execution, and potential for large-scale manufacturing. Numerous studies have reported the use of diverse botanical extracts. Biological and environmentally friendly approaches abstain from the use of hazardous chemicals during the preparation phase. There is a pressing need to develop ecologically benign biosynthetic techniques that can effectively mitigate the environmental risks posed by human activities. These methodologies depend on the utilization of bacteria, fungi, algae, and plants to generate AgNPs that possess optical, electrical, and antibacterial characteristics that are contingent upon their size and form. These phenomena are predicated on the bioreduction of Ag+ in aquatic environments [6-8] respectively.

*Avicennia marina*, a member of the Acanthaceae family, is predominantly distributed in subtropical and tropical climates of the Indo-West Pacific region. Secondary metabolites, including polyphenols, flavonoids, alkaloids, and tannins, have been demonstrated to be abundant in this substance. Owing to its numerous phytochemical classifications, this botanical specimen represents a viable selection for addressing a diverse range of health conditions. *A. marina* has been used as a traditional medicine for several generations. Leaves, such as those found in certain plants, have been employed for their medicinal properties in the treatment of ulcers, abscesses, and rheumatism [9].

The use of natural extracts as both reducing and stabilizing agents in the synthesis of AgNPs has garnered considerable interest in recent times owing to their potential applications in diverse domains, such as medicine, electronics, and environmental science. The plant under consideration is recognized for its diverse medicinal characteristics and presence of bioactive chemicals, rendering it a highly interesting candidate for the production of nanoparticles with potential biological activities. The objective of this study was to use *A. marina* leaf extract as a reducing and stabilizing agent for the synthesis of AgNPs. The use of botanical extracts in the process of nanoparticle synthesis presents numerous benefits, including diminished ecological repercussions, compatibility with living organisms, and the ability to exploit the inherent bioactive constituents of the plant for further biological functions. AgNPs produced using* A. marina* leaf extract possess distinct attributes and biological qualities, rendering them highly advantageous for a wide range of applications. Therefore, the primary objective of this study was to synthesize AgNPs using mangrove leaves from *A. marina* and evaluate their antibacterial properties against oral bacteria.

## Materials and methods

Preparation of *A. marina* leaf powder

A sample of *A. marina* mangroves was collected during the post-monsoon season of 2022 from Tuticorin District, located at latitude 8°44'57.6"N and longitude 78°11'07.1"E, in the state of Tamil Nadu. The sample was preprocessed through a cleaning and washing procedure using distilled water. The provided sample was subsequently subjected to a drying procedure in a hot-air oven, maintaining temperatures below 60 °C. Subsequently, the desiccated specimen was pulverized into a granular form using a mortar and pestle.

Preparation of aqueous extract of *A. marina* leaf powder

**Figure 1:**
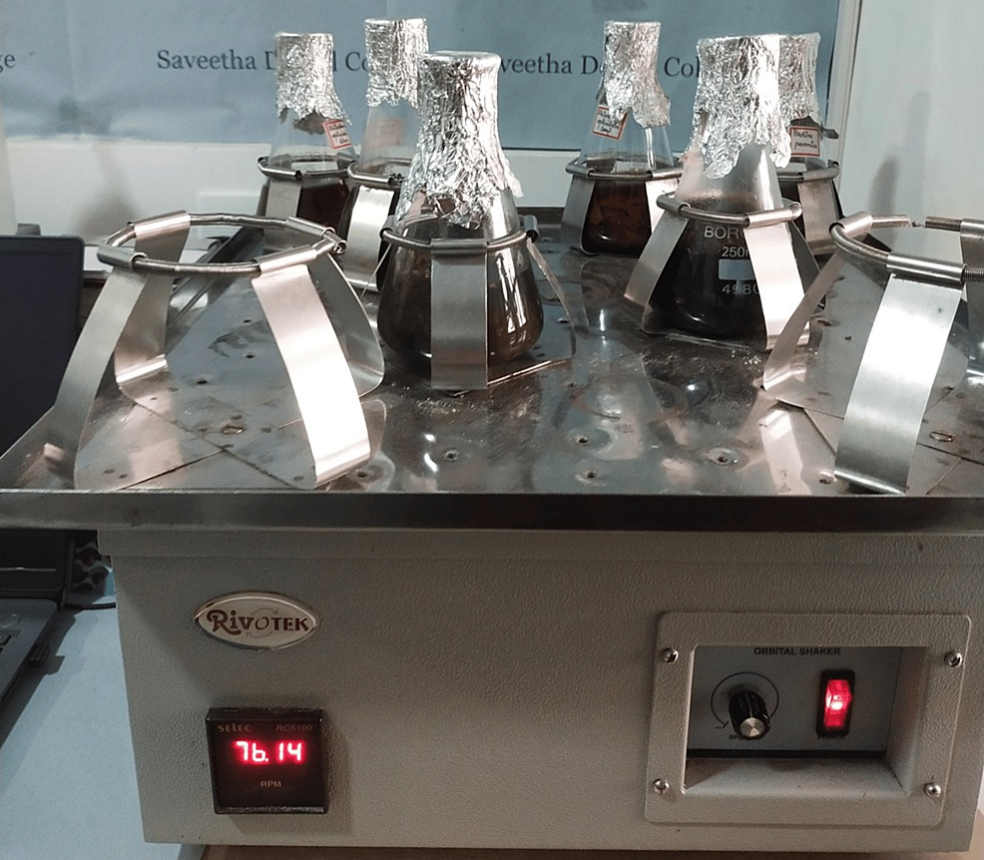
Preparation of aqueous extract from A. marina leaf powder using orbital shaker

Powdered sample (50 g) was mixed with 100 ml of distilled water in a conical flask. The mixture was placed on an orbital shaker (Figure 1) and agitated for 24 hours. The extracted material was subsequently passed through a muslin cloth and concentrated using a rotary evaporator to produce an unrefined extract.

Synthesis and characterization of AgNPs

An aqueous solution of AgNO_3_ was prepared by dissolving AgNO_3_ in double-distilled water. Subsequently, 100 ml of the AgNO_3_ solution was transferred into a conical flask. 5-10 mL of the aqueous extract was added dropwise under continuous stirring using an orbital shaker. The biosynthesized solution was observed visually and further investigated using a UV spectrophotometer at a wavelength of 420 nm. The material that underwent biosynthesis was centrifuged at 12,000 rpm. The pellets that were generated underwent separation and subsequent drying in a hot air oven. The dried nanoparticle sample was dissolved in 1 ml of ethanol and placed under sonication for 15 minutes. Subsequently, a 10 µl sample was applied onto aluminium foil and dried in an oven at 50 ºC. Fourier transform infrared spectroscopy (FTIR) and scanning electron microscopy (SEM) with energy-dispersive spectroscopy (EDS) analyses were performed as described by Sivaperumal et al. [10].

Antibacterial activity of AgNPs

The antibacterial activity of AgNPs from *A. marina* was assessed using the disc diffusion method [11]. Two distinct concentrations of AgNPs were impregnated into a 5 mm sterilized Whatman filter paper disc. Following inoculation, plates were incubated at ambient temperature for 24 hours. The zone of inhibition was measured in millimeters (mm) and the resulting AgNPs-produced growth inhibitory halos were measured. All the tests were performed in triplicate. Positive control discs containing 50 μl of tetracycline (1 mg/ml) were used.

## Results

Visual observation of AgNPs from *A. marina *leaf extract

**Figure 2:**
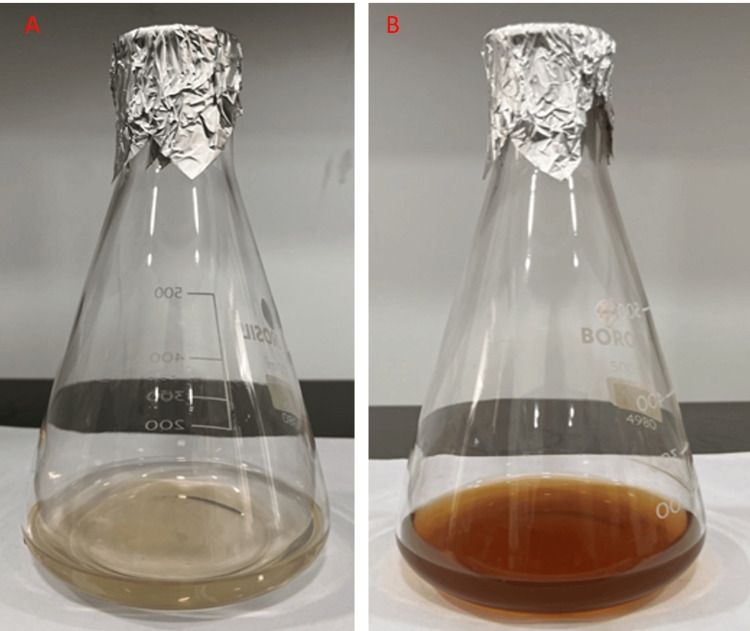
(A) Initial stage of silver nanoparticles (AgNPs) from Avicennia marina leaf extract, (B) Colour change after 24 hours

The solution containing AgNO_3_ and the aqueous extract was examined over a 24-hour period, revealing a noticeable darkening in color. This change in color indicated the successful formation of Ag nanoparticles (Figure 2).

UV visible spectroscopy of AgNPs from *A. marina *leaf extract

**Figure 3:**
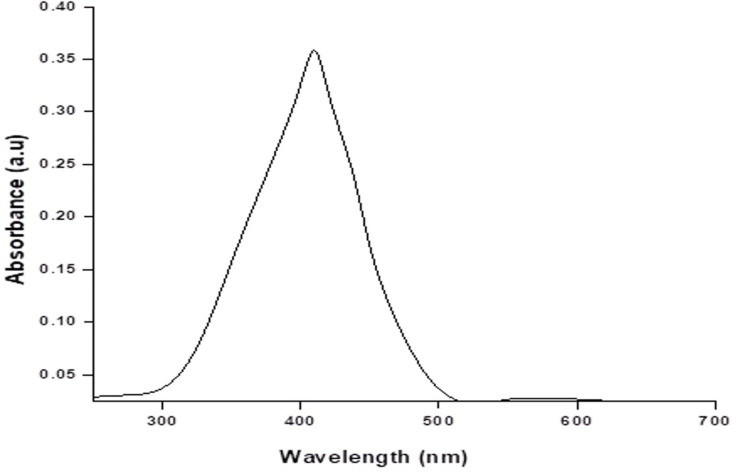
UV visible spectroscopy of synthesized silver nanoparticles (AgNPs) from Avicennia marina leaf extract

According to the UV spectrum graph, the Ag nanoparticles derived from *A. marina* leaves exhibited a peak absorbance of 0.35 at a wavelength of 420 nm (Figure 3).

FTIR analysis

**Figure 4:**
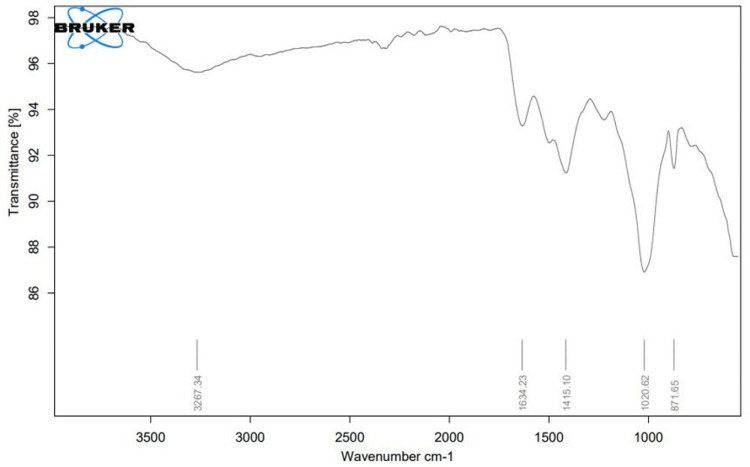
Fourier transform infrared spectroscopy (FTIR) spectrum of synthesized silver nanoparticles (AgNPs) from Avicennia marina leaf extract

In this study, FTIR operating within the frequency range of 500-3500 cm^−1^ was employed to identify the functional groups associated with the retrieval of silver ions and the coating agents used in the synthesis of AgNPs. FTIR analysis revealed distinct peaks at 871.65, 1020.62, 1415.10, 1634.23, and 3267.34 cm−1 (Figure 4). The characteristic peaks associated with specific functional groups are 871.65 cm⁻¹ strong peak corresponds to a strong bending vibration of the carbon-carbon double bond (C=C) in an alkene or vinylidene group. The 1020.62 cm⁻¹ medium peak is indicative of medium C-N stretching, characteristic of an amine group. In addition, 1415.10 cm⁻¹ strong peak corresponds to the strong stretching vibration of S=O bonds, typical of sulfates and 1634.23 cm⁻¹ strong peak is associated with the strong stretching vibration of the carbon-carbon double bond (C=C) in an alkene group. The 3267.34 cm⁻¹ strong, sharp peak indicates a strong and sharp stretching vibration of carbon-hydrogen (C-H) bonds in an alkyne group.

SEM with EDS analysis of synthesized AgNPs from *A. marina *leaf extract

**Figure 5:**
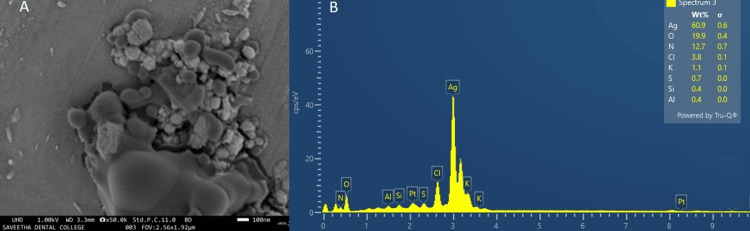
(A) Scanning electron microscopy (SEM) images of synthesized silver nanoparticles (AgNPs) from Avicennia marina leaf extract, (B) Energy-dispersive spectroscopy (EDS) analysis of synthesized AgNPs using Avicennia marina leaf extract showed the strong peak of silver

The SEM morphology of the image indicated agglomeration of the nanoparticles, with distinct particles ranging from 10 to 20 nm and dense rod-shaped particles (Figure 5A). EDS is a commonly used tool for identifying the presence of metals. The elemental composition of the biosynthesized AgNPs is shown (Figure 5B). A strong signal and maximum formation percentage were received at 42.7%, assigned to the element silver, and other peaks such as carbon, oxygen, and sodium are associated with Ag nanoparticles.

Antibacterial activity against oral bacteria

The antibacterial efficacy of AgNPs synthesized using green methods was assessed against three distinct oral bacteria: *Klebsiella *sp., *Staphylococcus aureus*, and *Streptococcus mutans.* The inhibition zone surrounding the disc placed on the back of the plate was measured (Table 1).

**Table 1:** Zone of inhibition by synthesized silver nanoparticles (AgNPs) from Avicennia marina leaves on oral bacteria: Klebsiella sp., Staphylococcus aureus, and Streptococcus mutans

Nanoparticle concentration (µg/ml)	Klebsiella sp. (mm) Mean ± SD	Staphylococcus aureus (MRSA) (mm) Mean ± SD	Streptococcus mutans (mm) Mean ± SD
50	8 ± 0.5	8 ± 0.2	7 ± 0.2
100	9.5 ± 0.5	9 ± 1	8 ± 0.25

**Figure 6:**
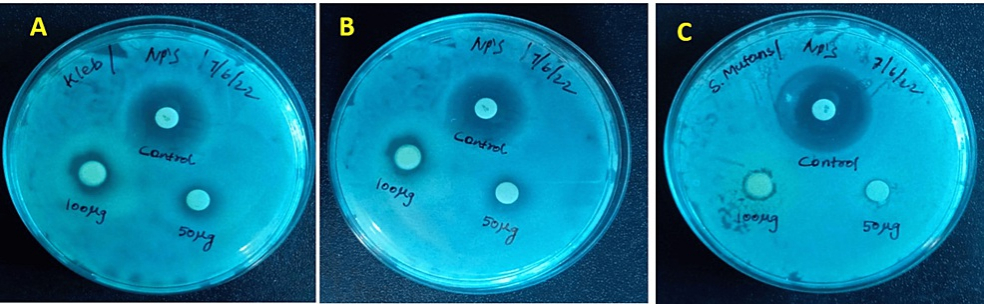
Antibacterial activity of silver nanoparticles (AgNPs) from Avicennia marina leaf extract: (3A) Klebsiella sp., (3B) Staphylococcus aureus, (3C) Streptococcus mutans

The nanoparticles exhibited significant antibacterial efficacy at two different doses, as evidenced by the presence of inhibition zones. The inhibition zones for *S. mutans*, *Klebsiella* sp., and *S. aureus* were measured at 8±0.25 mm, 9.5±0.5, and 9±1 mm, respectively, when exposed to a concentration of 100 ug/ml of AgNPs (Figure 6). Similarly, at 50 g/m, the inhibition zones of these bacteria were 7±0.2 mm, 8±0.5, and 8±0.2 mm, respectively.

## Discussion

Novel antibacterial agents with a range of biological, chemical, and physical properties are now being produced in the form of nanoparticles derived from plant extracts. The mangrove plant, *A. marina*, exhibited the most substantial production of AgNPs, as evidenced by the highest yield obtained from its leaf extract. This outcome could be attributed to the abundant presence of secondary metabolites, including polyphenols, flavonoids, and tannins [12]. The observed transition in solution color, specifically from yellow to dark brown, serves as empirical support for the biosynthesis of AgNPs. The presence of bioactive components in the extract is likely responsible for these observed effects [6]. The excitation of the surface plasmon vibration of the synthesized AgNPs could account for the observed color alteration [13]. In addition, AgNPs exhibited a distinct absorbance peak at 420 nm in the UV-visible spectrum, a property commonly observed in solutions containing AgNPs [14]. There has been significant interest in the potential use of nanoparticles in the development of antibacterial drugs. The inhibitory action of silver ions on bacteria has not been fully elucidated; however, the current understanding suggests that when exposed to Ag+, microbial DNA experiences a loss of reproductive capacity, while cellular proteins become inactive. Protein denaturation is observed in bacterial systems when Ag+ ions form complexes with functional groups present on proteins. Nanoparticles, akin to salt or bulk metals, possess supplementary characteristics, including an enhanced surface area and a gradual and consistent release of metal atoms [15,16].

There are several studies relevant to our current result to the identification of functional groups involved in the reduction of silver ions and serving as coating agents for silver nanoparticles was accomplished through FTIR analysis results revealed characteristic peaks at 3371.96, 1585.05, 1378.30, 1057.39, 823.39, and 521.07 cm⁻¹ [17] and similar peaks in *A. marina* mediated synthesized AgNPs peak at 831 to 3697 cm⁻¹ [18]. SEM analysis of AgNPs distinctly demonstrated clustered and irregular shapes, predominantly existing in aggregated forms with sizes ranging from 25 to 80 nm [18]. SEM images identified AgNPs ranging in size from 18 to 42 nm [19]. Likewise, previous studies on AgNPs showed the EDS spectrum revealed the presence of elements such as silver, chlorine, nitrogen, carbon, and oxygen in the nanoparticles produced from the leaf extract. Among these elements, Ag exhibited the highest percentage composition, constituting approximately 51.6% of the total elemental composition [20].

In a previous investigation, researchers successfully produced zinc oxide (ZnO) nanoparticles using a leaf extract derived from *Atalantia monophylla* exhibited a peak absorbance at 352 nm [21]. In a previous study, silver ions derived from *Mentha piperita* exhibited the greatest absorbance at 420 nm [22]. Another study demonstrated that nickel oxide nanoparticles synthesized using *A. marina* displayed a distinct peak in the UV absorption spectra at a wavelength of 297 nm [23]. The silver nanoparticles produced from *A. marina* had significant effects on three pathogens, namely *Klebsiella* sp. (9+1), *S. mutans* (9.5+0.5 mm), and *S. aureus* (8+0.25 mm). A disc diffusion assay demonstrated that *Klebsiella* sp. exhibited the largest zone of inhibition. The presence of *Streptococcus* mutations resulted in their subsequent occurrence, whereas the lowest reported incidence was associated with *S. aureus*. The potential antibacterial efficacy of nanoparticles derived from *A. marina* extracts against the tested pathogens could be attributed to various mechanisms, including denaturation of bacterial cell walls, inhibition of bacterial respiration, destabilization of microbial outer membranes, and depletion of intracellular ATP [24].

In previous investigations, it was observed that *E. coli* was more susceptible to AgNPs (6.25 ug/DL), *Enterococcus faecalis* was more resistant to AgNPs, and *S. aureus* showed higher activity (25 µg/DL) for gram-negative bacteria. Gram-negative bacteria possess a rigid outer membrane composed of lipids and lipoproteins, whereas gram-positive bacteria possess a substantial peptidoglycan layer. Despite the heightened motility and increased permeability barrier provided by AgNPs against gram-negative bacteria such as *S. aureus*. The antibacterial capabilities of AgNPs are contingent on their stability and structure [25,26]. In a previous investigation, silver nanoparticles generated using a biosynthetic method utilizing leaf extract derived from *A. marina* exhibited exclusive inhibitory properties against *S. aureus*. The average inhibition zone observed was measured to be 10.87±1.33 mm [27]. AgNPs produced through biosynthesis demonstrated significant antibacterial efficacy against all oral infections tested. The zone of inhibition of *S. mutans* at a volume of 50 µL was comparable to that of the standard antibiotic. A zone of inhibition comparable to that of conventional antibiotics was reported for *S. aureus* and *Pseudomonas* sp. at a volume of 25 µL. At a volume of 150 µL, AgNPs exhibited a zone of inhibition comparable to that of the conventional antibiotic for *E. faecalis* [28]. A zone of inhibition measuring 8-10 mm was observed at concentrations of 25, 50, and 150 μL. The antibiotic exhibited a maximal zone of inhibition of approximately 18 mm against the studied oral pathogenic microorganisms at all doses when exposed to graphene oxide nanoparticles [29]. In contrast, zinc nanoparticles derived from the same plant demonstrated inhibition zones against three distinct pathogens, namely *S. aureus*, *S. mutans*, and *Klebsiella* sp. The average inhibition zones measured 9.5±0.5 mm, 9±1 mm; 7.5±0.2 mm, 7±0.25 mm; and 7.5±0.2 mm, 7±0.25 mm, respectively. Another study revealed that the utilization of Ag/Fe2O3 nanoparticles at a concentration of 5 µg/ml had significant antibacterial effectiveness against *S. aureus*, leading to the formation of an inhibition zone of 22.3±0.57 mm [30].

The observed antibacterial activity of AgNPs derived from *A. marina* leaf extract can be attributed to the synergistic effect of both silver ions and phytochemical compounds that are naturally present in the extract. AgNPs possess a significant surface area that facilitates enhanced contact with the bacterial cells. The utilization of natural resources in the synthesis of AgNPs is considered ecologically sustainable and has the potential to mitigate the toxicity commonly associated with chemically synthesized nanoparticles. Additional research is warranted to examine the safety and long-term consequences of utilizing these AgNPs in oral contexts, with particular attention paid to the potential cytotoxicity and emergence of bacterial resistance. It is imperative to assess the stability and shelf life of AgNPs in order to ascertain their long-term effectiveness.

Limitations

The utilization of AgNPs in medicinal contexts gives rise to apprehensions regarding their biocompatibility and potential toxicity towards human cells and tissues. It is imperative to evaluate cytotoxicity and long-term effects on oral tissues. The oral cavity harbors a wide range of microorganisms that form a complex and diverse microbial ecosystem. The study should take into account the intricate nature of oral microbiota and conduct tests on nanoparticles against a more extensive spectrum of oral bacteria to assess their effectiveness. Most antibacterial investigations primarily use in vitro testing methodologies. However, in order to establish clinical significance, it is imperative to conduct in vivo investigations using animal models, followed by subsequent clinical trials using human subjects, in order to verify antibacterial efficacy.

## Conclusions

AgNPs were successfully synthesized using the aqueous leaf extract of *A. marina*. The process of characterizing them may be readily discerned, as the color undergoes a transition to a vivid red shade. Subsequent characterization was conducted using a UV spectrometer, and the peak was detected at a wavelength of 420 nm. The leaf extract of *A. marina* exhibited promising antibacterial properties, suggesting its potential utility in biomedical applications. In conclusion, the research conducted on the fabrication, partial characterization, and effectiveness against bacteria of silver nanoparticles derived from *A. marina* mangrove leaves has significant significance for both the preservation of the environment and the well-being of humans. It provides opportunities for the creation of new materials that could be used in dental healthcare and other areas. In summary, *A. marina* leaf extract for the production of AgNPs has potential as a viable antibacterial remedy for oral infections. However, further investigation is required to comprehensively understand the mechanisms by which they operate, ascertain their safety profiles, and explore their prospective use in the field of dental care and treatment.
